# Papillomavirus Immune Evasion Strategies Target the Infected Cell and the Local Immune System

**DOI:** 10.3389/fonc.2019.00682

**Published:** 2019-08-02

**Authors:** Chenhao Zhou, Zewen Kelvin Tuong, Ian Hector Frazer

**Affiliations:** ^1^Faculty of Medicine, The University of Queensland Diamantina Institute, Translational Research Institute, The University of Queensland, Brisbane, QLD, Australia; ^2^Molecular Immunity Unit, Department of Medicine, University of Cambridge, Cambridge, United Kingdom

**Keywords:** human papillomaviruses (HPVs), HPV-associated cancers, cervical cancer, HPV16, E7 oncoprotein, immune evasion, cancer immunotherapy, therapeutic vaccine

## Abstract

Persistent infection with human papillomavirus (HPV) initiates ~5% of all human cancers, and particularly cervical and oropharyngeal cancers. HPV vaccines prevent HPV infection, but do not eliminate existing HPV infections. Papillomaviruses induce hyperproliferation of epithelial cells. In this review we discuss how hyperproliferation renders epithelial cells less sensitive to immune attack, and impacts upon the efficiency of the local immune system. These observations have significance for the design of therapeutic HPV cancer immunotherapies.

## Introduction

Persistent infections by tumor-associated viruses, such as human papillomaviruses (HPVs), directly contribute to carcinogenesis through the expression of oncogenes (1). Oncogene proteins (oncoproteins) are involved in multiple regulation pathways, including, but not limited to disruption of cell-cycle checkpoints, controlling cell metabolism, modulation of the host immune response, and the induction of an immunosuppressive environment; features that collectively promote tumor initiation and survival. Cancer arising from persistence of HPV infection, although occurring only in a minority of HPV^+^ individuals, accounts for 4.6% of 14 million new cancer cases reported worldwide in 2012, and HPV-associated cancers comprise 29.1% of all 2.2 million infection-related cancers, including nearly 100% of cervical cancers (2, 3). Understanding the driving role of HPV in cancer development, and especially how persisting infection regulates the host immune system, is likely the key to developing effective cancer immunotherapies that fully eliminate virus infection. Moreover, studies of HPV-induced carcinogenesis may provide a general model for understanding other infectious agents that use similar principles to induce cancer.

## Epidemiology of HPV-Related Cancers

### HPV-Related Mucosal Cancers

More than 170 genotypes of HPV have been characterized, classified into 3 genera including Alpha-papillomavirus (α-HPV), Beta-papillomavirus (β-HPV), and Gamma-papillomavirus (γ-HPV) (4). However, only α-HPVs have been clearly linked to anogenital cancers, including cervical, anal, penile, vulvar, and vaginal cancers (5). Cervical cancer is the most common HPV-related cancer, with an estimated 570,000 new cases and 311,000 deaths in 2018 (2). The majority of cases are diagnosed in low- and middle-income countries (2). Two “high-risk” α-HPVs, HPV16 and HPV18, account for ~70% of cervical cancers in most epidemiological studies (6). HPV infection is also associated with non-anogenital cancers, with integrated HPV DNA being detectable in around 60% of oropharyngeal cancers (OPC) (7). Interestingly, although the incidence of other head and neck cancers has decreased in the last three decades, the incidence of OPC is increasing worldwide (7). This might be associated with the increased risk of sexually acquired oral HPV infection. Notably, if a woman is diagnosed with HPV^+^ cervical cancer, her partner has an increased risk of HPV^+^ OPC (8). In addition, HPV DNA has been detected in other cancers of the upper aerodigestive tract. Laryngeal papillomatosis is caused by low-risk HPV 6 and 11 (9). Despite the low oncogenic risk, recurrent laryngeal papillomatosis has a chance to develop into squamous cell carcinoma, which is likely caused by co-infection with high-risk HPV 16 and 18. A meta-analysis of 1,435 laryngeal cancers showed that HPV DNA was detectable in 24% of laryngeal cancers; Both low-risk HPV 6 and 11 and high-risk HPV 16 and 18 were commonly detected in these cancers (10). Notably, the prevalence of HPV in laryngeal cancer is highly variable in individual studies. This is largely due to inadequate sample sizes and differences in HPV detection methods, which need to be improved in further studies. Similarly, HPV infection has also been detected in esophageal cancer and its premalignant lesion, Barrett esophagus (11), despite that the frequency is highly variable across different studies. According to the estimation from meta-analysis, HPV infection is associated with 22.2% of esophageal squamous cell carcinoma, and with 35.0% of esophageal adenocarcinoma cases (12). The clinical relevance of HPV infection in treating esophageal cancers requires further investigation.

### HPV-Related Skin Cancers

While this review will primarily focus on the mechanisms associated with HPV16-associated cancer development in mucosal tissues, it is acknowledged that some HPVs can also contribute to cancers in cutaneous sites such as the skin. The majority of α-HPVs, including HPV16, infect mucosal sites, and promote cancer particularly at the junction between glandular and squamous epithelia, whereas β- and γ-HPVs and a small group of α-HPVs infect cutaneous squamous epithelia (13). DNA from cutaneous HPVs is commonly found in the skin of healthy individuals, with more than 40 β-HPV types and 50 γ-HPV types identified (14). Infection with β- and γ-HPVs can induce various skin lesions, but it is suggested that only β-HPVs are associated with induction of cancer (15). Non-Melanoma Skin Cancer (NMSC) is the most common cutaneous cancer in Caucasians with an increasing incidence worldwide (16). Exposure to ultraviolet radiation (UVR) is a major risk factor of NMSC. However, infection of β-HPVs has been postulated to act as a co-factor through a “hit-and-run” carcinogenesis hypothesis (17, 18). Unlike α-HPV associated cancers, which require ongoing HPV gene expression, skin cancers in patients that are genetically susceptible to HPV-associated skin cancers though mutations in the *EVER1/EVER2* genes do not always continue to express β-HPV genes (17, 19). Interestingly, β-HPV DNA is found more commonly in sun-exposed skin (20), implying increased β-HPV replication with UVR exposure, which may trigger the transcription of β-HPV DNA (21, 22). In addition, UVR may induce a local immunosuppressive environment that favors viral replication (23). Overall, β-HPV DNA is detected in 30–50% of NMSCs from immunocompetent patients (24), and 90% of NMSCs from immunosuppressed patients (25, 26). Although β-HPVs are present at very low viral loads in diseased skin (27), and are usually considered as a part of microbiota in healthy human skin (28), the deleterious effects of β-HPVs on the host DNA repair pathway and cell cycle regulation responding to UV exposure has been recently studied and reviewed (18). It remains to be discovered whether there is a *bona fide* etiological role of β-HPVs in NMSC initiation.

## Basics of HPV Biology

### HPV Genome and Life Cycle

HPVs are small, double-stranded DNA viruses. Their genome contains ~8,000 base pairs which form eight or nine open reading frames (29) that are designated as early (E) or late (L). The early genes, which encode the viral proteins E1–E7, have multiple roles in viral genome replication, cell cycle entry, immune modulation, and virus release. Their expression occurs throughout the viral life cycle but reduces during later stages of infection. In contrast, the late genes, which encode the viral capsid proteins L1 and L2, are highly expressed during later stages of infection (30).

HPVs exclusively infect human epithelial cells, and more specifically, basal keratinocytes. It has been suggested that infection requires epithelial wounding to allow viral access to the basal lamina, where basal keratinocytes are located (30, 31). Virus entry is initiated by the L1 and L2 proteins (32–35). After entering into basal keratinocytes, the viral genome is transported into the nucleus and is maintained as episomal DNA (36). The life cycle of HPV can be divided into a non-productive and a productive stage. In the non-productive stage, viral episomal DNA is amplified to 50–100 copies per cell in the nucleus of proliferative basal cells (37). Viral gene expression is minimal during this stage. The infected basal cells then leave the cell cycle and enter into the differentiation process, during which HPV begins its productive stage. In this stage, HPV significantly increases its DNA amplification and gene expression activity (38). In order to utilize the host's DNA replication machinery, which is suppressed in differentiating cells, HPV expresses the E1 helicase protein to facilitate access to single stranded viral DNA for replication, and the E6 and E7 oncoproteins to delay cell differentiation. E6 protein forms a complex with tumor suppressor protein p53 and recruits ubiquitination enzymes to degrade p53, preventing premature cell death. E7 protein on the other hand, disrupts the binding between retinoblastoma (Rb) protein and the E2F transcription factor, allowing the release of E2F to activate transcription of S-phase promoting genes in the host cells. The combination of E6 and E7 protein expression overrides cell cycle checkpoints and therefore allows HPV to replicate (39, 40). In the upper layers of the epithelium, HPV copy number is markedly increased up to thousands per cell. Viral capsid proteins are synthesized and assembled in the terminally differentiated cells. The assembled capsid proteins form a coat that encapsulates viral genomes, and HPV is then shed from differentiated infected epithelial cells (30, 41).

### HPV Pathogenesis

HPV-associated carcinogenesis has been extensively studied in the human genital tract, where around 30 strains are known to cause infection. These HPVs can be divided into “high-risk” genotypes (e.g., HPV16 and 18) that are associated with genital cancers, and “low-risk” genotypes (e.g., HPV6 and 11) that are typically found within genital warts or normal genital epithelium (30). HPV infections of the genital tract are sexually transmitted, and most individuals that partake in sexual activity will become infected by at least one genital HPV type in their lifetime. High risk HPV infection in the female genital tract initially causes low-grade squamous intraepithelial lesions (LSIL), also known as cervical intraepithelial neoplasia grade 1 (CIN 1). These lesions, within which viral replication occurs, show only mild dysplastic changes. They are usually cleared by the immune system within 1 year (42). However, if lesions persist, they may progress into high-grade squamous intraepithelial lesions (HSIL), also known as CIN 2 (moderate dysplasia) or CIN 3 (severe dysplasia and carcinoma *in situ*) (42, 43). When patients present with HSIL in the cervix and are left untreated, the risk of progression to cervical cancer is substantially escalated. It has been estimated that HSILs can persist for several decades before progressing to cervical cancer. The risk for developing invasive cervical cancers in HSIL patients is ~20% at 5 years, and increases to 50% at 30 years (42, 44).

Persistent infection by high-risk HPVs is the greatest risk factor for cervical cancer development (42, 45). This is largely due to the complementary function of high-risk E6 and E7 oncoproteins in infected cells. While low-risk HPVs also produce E6 and E7 proteins, these interact with cellular proteins in different ways compared with high-risk E6 and E7 proteins. Both low- and high-risk E6 proteins are able to bind with p53. However, only high-risk E6 proteins contact with the core domain of p53, which is essential to recruit ubiquitin ligase and mark p53 for degradation (46). Similarly, both low- and high-risk E7 proteins are capable of interacting with tumor suppressor Rb proteins. However, high-risk E7 proteins have a much higher affinity for Rb compared to low-risk E7 proteins, and it has been suggested that this high affinity is essential to disrupt interactions between Rb and E2F (47). Overall, high-risk E6 and E7 proteins are highly effective at disrupting cell-cycle checkpoints. This results in genomic instability and high risk of transformation in infected cells that combine to drive cervical cancer progression.

The integration of high-risk HPV genomes into the host genome is another key event for HPV-associated carcinogenesis. It has been suggested that high-risk, but not low-risk, HPV E6 and E7 proteins facilitate HPV DNA integration into the host genome (48). This could be a consequence of the increased chromosome rearrangement events in high risk E6- and E7-expressing cells. In addition, high-risk E6 and E7 proteins may have increased potential to directly disrupt normal DNA repair pathways (48). High-risk HPV genome integration may lead to deletion and/or mutation of both host and viral genes (49–52). Notably, the HPV16 E1 or E2 open reading frame is often disrupted during genome integration (53). The expression of E1 and E2 genes in the early phase of infection play essential roles in negatively regulating the expression of E6 and E7 genes. The disruption of E1 and E2 genes will therefore lead to loss of control of E6 and E7 expression, which further promotes the progression to cancer.

In summary, pathogenesis of high-risk and low-risk HPVs fundamentally differs. Low-risk HPVs have evolved a life cycle that is characterized by rapid production of virus progeny and formation of large productive lesions to maximize their transmission to a new host. Low-risk HPV E6 and E7 proteins play critical roles during viral life cycle, but they exhibit low transforming activities and do not contribute to genomic instability. In contrast, high-risk HPVs have evolved to maintain a low copy number in infected cells and can persist for decades, without causing clinical disease. The transforming capacity of high-risk HPVs reflects their need for persistence in non-cycling, differentiated epithelial cells. Moreover, transformed cells may impact the local immune environment and increase the likelihood that high-risk HPVs will escape from immune attack. Relevant immune evasion mechanisms are discussed in the following sections.

## Immune Evasion Mechanisms of HPV in Keratinocytes

Most individuals will clear HPV-associated lesions within 1–2 years (42, 43), indicating that the host immune system is capable of controlling HPV infection. On the other hand, despite the onset of an immune response, HPV-associated lesions can persist for months if not years before regression (54). Furthermore, there is also a delay of 6–12 months before anti-HPV antibodies can be detected in infected individuals (55). This indicates that HPVs employ intrinsic mechanisms to reduce the efficiency of host immune surveillance, which may increase the susceptibility toward persistent HPV infection when individuals are exposed to additional risk factors. For example, immunosuppressed patients, such as renal transplant recipients (56, 57) and patients infected with human immunodeficiency virus (HIV) (58), have a higher prevalence of persistent HPV infection. Individuals with certain major histocompatibility complex (MHC) alleles (e.g., HLA-DQB1^*^0602 and HLA-DRB1^*^1501) are also more susceptible to persistent HPV infection and have an increased risk of developing cervical cancer (59). While the biological mechanisms underlying the protective and risk associations between the HLA alleles, HPV antigens and HPV-related cervical cancer remains to be discovered, it could be conceivably linked that HPV antigens presented by MHC molecules have variable affinities, which may or may not be immunogenic enough to result in effective priming of HPV-specific immune cells in protected vs. at risk individuals. Moreover, other coincidental factors, such as unpredictable DNA replication errors associated with persistent HPV infection, are likely to initiate progression from chronic inflammation into cancers.

**Figure 1:**
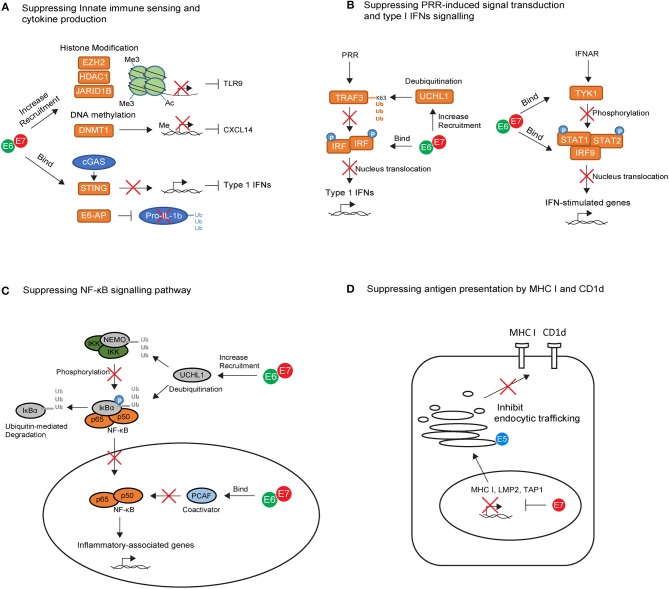
The key intrinsic immune evasion mechanisms exploited by HPV oncoproteins in keratinocytes (KCs). **(A)** High-risk HPV E6 and E7 recruit histone modifying enzymes (EZH2, HDAC1, and JARID1B) and DNA methylation enzyme (DNMT1) to suppress TLR9 and CXCL14 transcription. E6 and E7 also bind to the adapter protein STING and ubiquitin ligase E6-AP, leading to downregulation of cGAS–STING cytosolic DNA sensing system and degradation of pro-IL-1β. **(B)** High-risk HPV E6 and E7 block pathogen recognition receptors (PRR) signal transduction cascades by upregulating the deubiquitinating enzyme Ubiquitin C-Terminal Hydrolase L1 (UCHL1) to inhibit TNF-receptor-associated factor 3 (TRAF3) activation, and by binding to interferon regulatory transcription factor (IRF) to prevent its transcriptional activity in the nucleus. E6 an E7 also interfere with IFN-α/β receptor (IFNAR) signaling pathway by binding to tyrosine kinase 2 (TYK2) to hamper phosphorylation of STAT1 and STAT2, and by interacting with IRF9 to prevent its binding to phosphorylated STAT1 and STAT2 for activating IFN-stimulated genes. **(C)** High-risk HPV E6 and E7 prevent the nuclear translocation of NF-κB via upregulation of UCHL1. E6 and E7 are also capable of binding to P300/CBP-associated factor (PCAF), a coactivator of NF-κB in the nucleus, thereby downregulating the NF-κB signaling pathway. **(D)** High-risk HPV E7 can interact with the major histocompatibility complex (MHC) I promoter, leading to repression of MHC I, LMP2, and TAP1 gene. In contrast, E5 reduces MHC I and CD1d expression by blocking the transport of MHC I and CD1d proteins to the cell surface via its interactions with host proteins in the Golgi complex and ER.

HPV can also evade immune detection via minimization of antigen production during the vegetative virus life cycle. In the early phase of infection, HPVs express a low abundance of proteins, which are promptly translocated to the cell nucleus (60), thus minimizing presentation to the host immune system. In the late phase of infection, HPVs increase the expression of highly immunogenic capsid proteins (61), however, these proteins are shed off quickly from the outer layer of the epithelium, which has a low density of antigen presenting cells. These are referred to as passive immune evasion strategies. In addition, HPVs utilize aggressive immune evasion strategies, determined by the expression of HPV oncoproteins E6 and E7. As will be discussed below, these oncoproteins take advantage of their high binding affinity to cellular immune regulator proteins, to block immune-related gene expression and immune signaling pathways in infected keratinocytes (Figures 1A–D). The impairment of immune responses in infected cells affects their capacity to alert regional immune cells, resulting in an overall immunosuppressive environment that promotes cancer development.

### High-Risk HPV Oncoproteins Impair the Immune Alarm Function of Keratinocytes

The immune-modulatory role of high risk HPV proteins has been established mainly through the *in vitro* analysis of HPV oncogene-expression in keratinocytes. Keratinocytes act as immune sentinels and express pathogen recognition receptors (PRRs), which recognize pathogen-associated molecular patterns presented by the virus (62). For example, Toll-like receptor 9 (TLR9) can specifically recognize viral double-stranded DNA molecules and trigger downstream inflammatory signaling cascades (62). HPV oncoproteins use several strategies to suppress TLR9 expression in keratinocytes (Figure 1A). HPV38 E7 recruits histone modifying enzyme EZH2 to the TLR9 promoter region, resulting in histone methylation and repression of TLR9 transcription (63). Similarly, HPV16 E7 can recruit histone deacetylase HDAC1 and histone demethylase JARID1B to the regulatory region of the TLR9 promoter, leading to downregulation of TLR9 expression (64). In addition to TLR9, viral DNA can be detected by the cytosolic DNA sensor, cyclic guanosine monophosphate (GMP)-adenosine monophosphate (AMP) synthase (cGAS). Viral recognition via cGAS activates downstream signaling through the adapter protein STING. HPV18 E7 has been recently found to bind and block STING via its LXCXE motif (Figure 1A), leading to the reduced production of pro-inflammatory cytokines in the keratinocyte (65).

A few studies have attempted to address the association between TLR9 expression and cervical cancer in humans but there is a lack of consensus in the literature. For example, cervical epithelial cells expressing high level of TLR9 were found to be less susceptible to HPV infection compared to those with low TLR9 expression (66). In contrast, high TLR9 expression was reported in cervical cancer patients in other studies (67, 68). It is plausible that TLR9 plays dual roles in shaping the initial host response at the earliest point of HPV infection, whilst also driving tumor-associated inflammation during the chronic stage of HPV infection, thereby contributing to cancer development. The association between the cGAS-STING pathway and HPV-associated cancers has been under-studied to date. However, a recent study revealed that a special form of polymorphism in the cGAS gene is associated with genetic susceptibility to cervical precancerous lesions including LSILs and HSILs (69). This implies the contribution of cGAS-STING pathway in HPV clearance in infected individuals.

Upon viral recognition, PRRs transduce intracellular signals to initiate the production of pro-inflammatory cytokines including type I Interferons (IFNs), mainly IFNα and IFNβ. The success of PRR signal transduction cascades requires the activation of TNF-receptor-associated factor (TRAF) and subsequent phosphorylation of interferon regulatory transcription factor (IRF). Phosphorylated IRFs dimerize and translocate to the nucleus in which point the production of type I IFNs is initiated (70). *In vitro* studies have shown that HPV oncoproteins interfere at several points in this signaling cascade (Figure 1B). For example, HPV16 E6 can bind to IRF3 and therefore prevent its transcriptional activity in the nucleus (71), while HPV16 E7 blocks IFNβ transcription by binding to IRF1 and recruiting histone deacetylases to the IFNβ promotor site (72). In addition, HPVs also upregulate the deubiquitinating enzyme Ubiquitin C-Terminal Hydrolase L1 (UCHL1) to deubiquitinate K63-linked polyubiquitin chains from TNF-receptor-associated factor 3 (TRAF3), resulting in the inhibition of TRAF3 activation (73). The production of type I IFNs can act as positive feedback loop to enhance IFN-stimulated gene expression in the keratinocyte itself as well as in neighboring cells through IFN-α/β receptor (IFNAR) signaling pathways (70). *In vitro* studies have also revealed that HPV oncoproteins use several strategies to interfere with IFN-receptor pathways (Figure 1B). They can bind to tyrosine kinase 2 (TYK2) to hamper phosphorylation of STAT1 and STAT2 (74), the transcription factors which are required for IFN-stimulated gene transcription. In addition, high-risk HPV E6 directly impairs STAT1 transcription and translation (75–77), whereas E7 interacts with IRF9, preventing IRF9 binding to phosphorylated STAT1 and STAT2 and forming a complex for nucleus translocation (78, 79). This may explain why clinical studies in the past few decades have highlighted the limited effect of IFN therapy in the treatment of HPV genital infections (80). Interestingly, the response rate to IFNα in patients with low-risk HPV infection is higher than those with high-risk HPV infection (81), and biopsy samples derived from cervical cancer patients showed downregulation of type I IFN expression compared with tissue from normal individuals (82). This suggests that high-risk HPVs are more efficient at promoting host resistance to IFN signaling.

The downregulation of NF-κB pathway is another critical, but perplexing, strategy used by HPV oncoproteins for immune evasion (Figure 1C). NF-κB play a key role in immune surveillance by promoting the cellular expression of genes involved in antigen presentation, IFNs, β-defensines, and cytokine production (83). There is evidence supporting HPV16 E6 and E7 inhibition of NF-κB activity in keratinocytes cultured from the human cervical transformation zone, in which most cervical cancers arise (84). Furthermore, high-risk HPV-infected keratinocytes upregulate UCHL1, which effectively suppresses downstream anti-viral responses such as production of type 1 IFNs, as well as suppression of p65 phosphorylation, and thereby nuclear translocation of p65, via degradation of NEMO, a regulatory subunit of the inhibitor of kappaB kinase (IKK) complex, and via promoting stabilization of IκBα, an inhibitor of the NF-κB complexes (73). Additionally, high-risk HPV E6 and E7 are capable of binding to coactivators of NF-κB in the nucleus, thereby downregulating the NF-κB signaling pathway (85–87). Overall, NF-kB downregulation results in impaired anti-HPV activity in the infected keratinocytes, promoting persistence of HPV infection. However, the NF-κB pathway is paradoxically highly activated in HSILs and cervical cancers (88), suggesting that NF-κB also plays a tumor-promoting role during cervical carcinogenesis. Given that high-risk HPV E6 and E7 inhibited NF-κB pathway, activation of NF-kB in HSILs and cervical cancers is likely to be mediated by signals derived from the local protumorigenic microenvironment, such as tumor-associated inflammatory cells and fibroblasts (83). It is proposed that high NF-kB activity contributes to carcinogenesis by variety of mechanisms. Firstly, NF-kB pathway can stimulate expression of protumorigenic genes involved in cell proliferation, immortalization, vascular endothelial growth factor (VEGF)-dependent angiogenesis and metastasis (83). On the other hand, during chronic stage of HPV infection, the pro-inflammatory role of NF-kB pathway can inhibit tumor growth, but also promote more aggressive tumors which escape immune destruction, a process called cancer immunoediting (89). In this context, the protumorigenic role of NF-kB activity becomes dominant.

Using similar strategies, HPVs are capable of regulating multiple other immune signaling pathways in keratinocytes. Recently, the production of interleukin (IL) 1-β in keratinocytes has also been found to be downregulated by HPV oncoproteins. HPV16 E6 forms a complex with ubiquitin ligase E6-AP and the tumor suppressor p53, leading to the degradation of pro-IL-1β and impairment of IL-1β formation (90) (Figure 1A). Indeed, clinical studies of human biopsy samples revealed a progressive loss of IL-1β gene and protein expression from normal epithelium to CIN lesions, and from CINs to cervical tumors (90). In addition to IL-1β suppression, HPV oncoproteins were also found to associate with DNA methyltransferase 1(DNMT1), leading to hyper-methylation of the CXCL14 promoter (91) (Figure 1A). Downregulation of CXCL14 has also been observed in HPV-associated cancers (91). It has been suggested that CXCL14 may play important roles in angiogenesis inhibition (92) and in promoting the recruitment of antigen-presenting cells (APCs), natural killer (NK) cells, and T cells (93), thereby preventing HPV-associated cancer progression.

### High-Risk HPV Oncoproteins Prevent Keratinocytes From Being Recognized by Adaptive Immunity

Killing of infected cells by antigen-specific cytotoxic T lymphocytes (CTLs) is a highly effective and specific mechanism to eliminate virus infection in the host. CTL-mediated killing is dependent on the presentation of pathogen-derived peptides by MHC class I molecules on the surface of keratinocytes. It has been reported that around 30% of cervical cancers have reduced MHC I expression (94, 95). Downregulation of MHC I in these cancers also correlates with reduced levels of transporter associated with antigen processing (TAP), a protein complex that transports cytosolic peptides into the endoplasmic reticulum (ER) (96). Interestingly, the expression of MHC II molecules is frequently upregulated in cervical tumor cells (96, 97). This suggests a potential interaction between neoplastic keratinocytes and HPV-specific CD4^+^ T cells, which may promote tumor progression.

Several mechanisms may combine to lead to MHC I downregulation following HPV infection (Figure 1D). For example, high-risk HPV E7 can interact with the RXR-beta binding motif of the MHC I promoter and recruit histone deacetylases to the promoter site, leading to repression of MHC I gene expression (98–102). Using similar strategies, E7 represses expression of antigen processing machinery components LMP2 and TAP1 (98, 99), leading to impairment of peptide production and transportation in infected cells. In contrast to E7, E5 reduces MHC I expression by blocking the transport of MHC I to the cell surface via its interactions with host proteins in the Golgi complex and ER (103–105). E5 also downregulates the surface expression of CD1d molecules on keratinocytes (106) (Figure 1D). The loss of CD1d has been observed in cervical cancer lesions (106). CD1d is an antigen presenting molecule essential for the activation of natural killer T (NKT) cells. The loss of CD1d may downregulate NKT cell-mediated anti-viral responses, which is likely to impact upon the very early stages of immune activation following HPV infection. However, downregulation of CD1d in cervical cancer lesions may not be mediated by E5, as E5 expression is often inactivated in tumor cells as a consequence of viral genome integration. Therefore, other mechanisms may play important roles and need further clarification.

Overall, there is accumulating evidence that HPV oncoproteins target multiple immune-associated pathways in keratinocytes. The disruption of these pathways collectively promote the immune evasion of HPV in the early stage of infection, and will induce susceptibility toward persistence of HPV infection and progression to HPV-associated cancers. As will be discussed further below, the impairment of multiple immune-associated pathways in keratinocytes may lead to inefficient activation and function of other immune components, resulting in a compromised immune response network against HPV.

## Immune Evasion of HPV by Modulating the Immune Network

### Impaired Function of the Innate Leukocytes in HPV-Associated Cancers

In addition to keratinocytes, the innate immune system also includes bone marrow-derived leukocytes. These innate leukocytes are recruited to the peripheral tissues and activated in response to signals derived from local microenvironments. Together with keratinocytes, they promote a cytokine-mediated pro-inflammatory environment, which is essential in triggering the adaptive immune response to eliminate infected cells. In HPV-associated cancers, however, many of these innate leukocytes were observed to display compromised or regulatory functions. They act as one of the major factors leading to an inefficient adaptive immune response and eventually immune evasion by HPV.

#### Professional Antigen Presenting Cells

**Figure 2:**
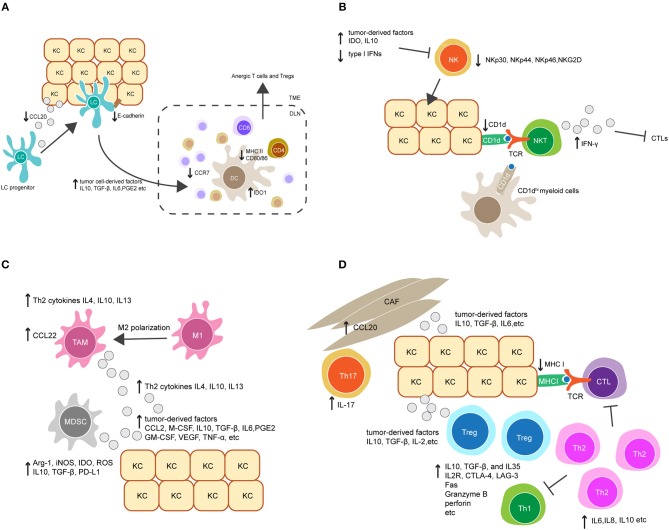
Crosstalk between keratinocytes (KCs) and immune cells orchestrates immunosuppression in HPV-associated tumor microenvironment. **(A)** Downregulation of the chemokine CCL20 and cell adhesion molecule E-cadherin in HPV-infected KCs reduces Langerhans cells (LCs) infiltration in HPV-associated tumor. LCs and migratory dendritic cells (DCs) in tumor microenvironment also display immature or regulatory phenotypes and have reduced migratory capacity to secondary lymphoid tissue, characterized by downregulation of MHC II, CD80, CD86, and the chemokine receptor CCR7, and upregulation of indoleamine 2, 3-dioxygenase 1 (IDO1). This is probably mediated by tumor cell-derived immunosuppressive factors, such as IL-10, TGF-β, IL-6, and prostaglandin E2 (PGE2). **(B)** Upregulation of tumor-derived factors and downregulation of type I IFNs in HPV-associated tumor suppress NK cell activation and its killing capacity against tumor cells. On the other hand, downregulation of CD1d on KCs dampens NKT cell activity. CD1d^hi^ myeloid cells in tumor environment might contribute to an alternative source of CD1d, leading to the activation of immunosuppressive IFN-γ-producing NKT cells. **(C)** The accumulation of tumor-associated macrophages (TAMs) and myeloid-derived suppressor cells (MDSCs) is mediated by a variety of tumor-derived factors, such as the chemokine CCL2, macrophage colony-stimulating factor (M-CSF), IL10, TGF-β, IL6 and PGE2. Additionally, Th2-associated cytokines promote TAM differentiation by inducing a phenotypic switch from M1 to M2. TAMs produce Th2-associated cytokines to promote Th2 cell differentiation, and secret the chemokine CCL22 to recruit Tregs. Similarly, MDSCs inhibit the effector immune response by producing a broad range of suppressive molecules, such as arginase 1 (Arg-1), inducible nitric oxide synthase (iNOS), IDO, reactive oxygen species (ROS), IL10, TGF-β, and PD-L1. **(D)** Tumor-derived factors promote the accumulation of Tregs and a shift from a Th1 toward a Th2 response in local microenvironment. In addition, the recruitment of Th17 cells is increased by CCL20 secretion in cancer-associated fibroblasts (CAFs). Collectively, these modulated responses might in part contribute to downregulation of CTL responses.

Langerhans cells (LCs) are specialized APCs resident in the epidermis as a part of immune sentinels. Upon antigen recognition, their migration to secondary lymphoid tissues enables the priming of adaptive immune cells. Interference with LC trafficking in and out of the epidermis can therefore assist in immune evasion by HPV (Figure 2A). In HPV-associated cancers, low infiltration of LCs in tumors have been associated with disease severity in HPV16^+^ cervical and head and neck cancer lesions (107–109). This is possibly due to downregulation of CCL20, a chemokine which attracts LC to sites of inflammation (110). HPV16 E6 and E7 expression promote CCL20 downregulation, which is thought to be achieved through the capacity of E6 and E7 to inhibit the NF-κB signaling pathway (111). In addition, HPV-associated tumors have reduced expression of E-cadherin compared to healthy epidermis (112). E-cadherin is an adhesive molecule that allows LCs to remain in the epidermis to enable viral antigen uptake (112). It has been suggested that HPV16 E7 is responsible for suppression of E-cadherin expression by inducing methylation of the E-cadherin promoter (113). Similarly, CCR7 expression on migratory dendritic cells (DCs) is downregulated in HPV-associated tumors (114), resulting in a reduction of DC homing to secondary lymphoid tissue (115). Altogether, these data imply impairment of LC trafficking in HPV-infected epithelium. The loss of LC presenting viral antigen might partially contribute to the poor priming of effector T cells. Targeting LC trafficking therefore warrants closer study to define whether therapeutic intervention would restore anti-HPV immune responses in patients with HPV-associated cancers.

In addition to impacted trafficking, APCs also display immature phenotypes in HPV-infected epithelium, characterized by downregulation of cell surface MHC molecules and co-stimulatory molecules including CD80 and CD86 (116). Downregulation of these molecules reduces the capacity of DCs to prime antigen-specific T cells (Figure 2A). DC maturation is inhibited in many cancers, as a consequence of secretion of tumor cell-derived immunosuppressive factors, such as IL-10, transforming growth factor (TGF)-β, IL-6, prostaglandin E2 (PGE2) and granulocyte-macrophage colony-stimulating factor (GM-CSF) (117–119). Indeed, these factors have been demonstrated to be important modulators for the establishment of an immunosuppressive environment in HPV-associated cancers (120, 121). Further studies are needed to investigate the impact of these factors on DC maturation in HPV-infected epithelium. Notably, several studies have tested whether promoting LC maturation may have potential as a therapeutic strategy to combat HPV infection. For example, Polyinosinic:polycytidylic acid (Poly I:C) and the cell-derived cytokine-based biologic IRX-2 are two immunostimulants that were able to upregulate MHC and costimulatory molecule expression on LCs pre-exposed to HPV16. Furthermore, Poly I:C and IRX-2 were also shown to enhance LC migration to secondary lymphoid tissues by upregulating CCR7 expression on LCs. These modulating effects allowed LCs to restore their capacity to induce CD8^+^ T cell immune responses against HPV16-derived peptides (122, 123). Current studies are further evaluating the translational potential of Poly I:C or IRX-2 in treating HPV-associated cancers.

Recently, a regulatory APC has been identified in mouse skin transgenic for HPV16 E7. These regulatory cells were found in the dermis and expressed high level of indoleamine 2, 3-dioxygenase 1(IDO1) (124). This is consistent with clinical observations that HPV^+^ cervical lesions have a high level of IDO1 expression (125). IDO1 is an enzyme that negatively regulates anti-tumor immunity (126). HPV16 E7-transgenic mouse skin with infiltrating dermal IDO1^hi^ APCs was not rejected when grafted onto non-transgenic recipient mice, whereas the inhibition of IDO1 promoted rejection (124). This implies that IDO1 produced from these APCs might contribute to immune tolerance in HPV16 E7-expressing skin. Further studies are necessary to investigate the origin and function of these IDO1^hi^ APCs. It is likely that they are capable of competing with other immunogenic APC populations for T cell priming, leading to the induction of both anergic T cells and regulatory T cells (Tregs) (Figure 2A).

#### NK and NKT Cells

NK cells are innate immune cells that can recognize and kill virus-infected cells which have down-regulated surface MHC I molecules and are resistant to CTL-mediated killing. The activity of NK cells was impaired in patients with persistent HPV infection and cervical cancer, as evidenced by the downregulation of several NK-activating receptors including NKp30, NKp44, NKp46, and NKG2D (127). Furthermore, recent studies indicate that the impairment of NK cell activity in HPV^+^ cervical lesions is mediated by the upregulation of tumor-derived immune checkpoint molecules such as IDO (128, 129). As discussed above, IDO^+^ DCs can be found within persisting HPV16 E7 expression in skin. IDO expression within the tumor environment is also reported to be upregulated by activated macrophages, and APC, as well as tumor cells (130). This suggests that these cells may interfere with NK cell activity in HPV^+^ cervical lesions. In addition, upregulation of IL-10 and downregulation of type I IFNs in HPV^+^ lesions was found to suppress NK cell differentiation and activation (131, 132). Altogether, these findings suggest the important role for tumor-induced immunosuppressive factors in modulating NK cell activity in the tumor environment (Figure 2B).

NKT cells are a subset of T cells that share properties of both T cells and NK cells. The best characterized NKT cell expresses a semi-invariant TCR and releases high amounts of pro-inflammatory cytokines upon recognition of CD1d molecules, which present lipid antigens on the surface of APCs and tumor cells (133). For these reasons, NKT cells are often regarded as innate immune cells. There are a few studies addressing the role of NKT cells in HPV^+^ precancerous and cervical cancer lesions. CD1d is downregulated in HPV16-infected cells *in vivo* and *in vitro* as a result of HPV16 E5 expression (106), which might help HPV-infected cells evade protective NKT cell activity (134). On the other hand, the immunosuppressive role of NKT cells has been described in the HPV16 E7 transgenic mouse model (135, 136). Infiltration of NKT cells was observed in grafted HPV16 E7-transgenic mouse skin but not in non-transgenic skin. These NKT cells were characterized by high levels of IFN-γ production, which was shown to suppress the proliferation and cytotoxicity of CD8 T cells, thereby inhibiting HPV16 E7-expressing skin graft rejection. In human studies of HPV^+^ high-grade lesions, the accumulation of IFN-γ-producing NKT cells has also been observed (137). This implies that IFN-γ-producing NKT cells are paradoxically immunosuppressive and may contribute to HPV-associated carcinogenesis (Figure 2B). On further exploration of the origin and function of these NKT cells, a population of CD11c^+^ F4/80^hi^ CD1d^hi^ myeloid cells was observed to be recruited into grafted E7 transgenic skin (135, 136). Given that CD1d expression is downregulated in HPV-infected keratinocytes, it is possible that these CD1d^hi^ myeloid cells contribute to an alternative source of CD1d which leads to the activation of immunosuppressive NKT cells in the local tissue environment (Figure 2B). Moreover, IFN-γ is known as an inducer of IDO1 expression (124). Therefore, increased IDO1 expression observed in the HPV-associated tumor environment might be induced by IFN-γ-producing NKT cells, resulting in a feed-forward loop of immunosuppression. Interestingly, the regulatory IDO1^hi^ DCs found in HPV16 E7-transgenic mouse skin expressed a high level of IFN-γ receptor (135, 136). This further supports a potential interaction between NKT cells and DCs in promoting HPV-associated immunosuppression.

#### Regulatory Innate Leukocytes

Regulatory innate leukocytes are naturally found at inflammatory sites and act as a negative feedback control for effector immune responses. In a virus-associated tumor environment, the recruitment of these regulatory cells is often increased, which can enhance viral immune evasion. Tumor-associated macrophages (TAMs), for example, frequently present with a M2-polarized phenotype, and the presence of TAMs has been correlated with tumor progression and poor prognosis in cervical cancer patients (138, 139). High grade HPV^+^ cervical lesions were characterized by increased macrophage infiltration, in which TAMs were found to be the predominant macrophage population (139, 140). The underlying mechanism for the recruitment of TAMs in HPV-associated lesions is not fully understood (Figure 2C). It has been suggested that TAMs differentiate from monocyte-derived macrophages following exposure to a variety of tumor cell-derived soluble factors. These factors include the chemokine CCL2 and the macrophage colony-stimulating factor (M-CSF), and several immunosuppressive modulators such as TGF-β, IL-10, IL-6, and PGE2 (141), which have been found to be upregulated in HPV-associated tumors. In addition to tumor cell-derived factors, T helper 2 (Th2) cytokines such as IL-4, IL-10, and IL-13 also promote the differentiation of TAMs, whereas Th1 cytokines have an inhibitory role (142). Interestingly, it has been suggested that the interaction between Th1 cells and TAMs can induce the repolarization of TAMs into M1 macrophages, an anti-tumor type of macrophage (143). This reveals the functional plasticity of macrophages in response to different local environmental factors. Blockade of TAM differentiation and targeting of macrophage polarization to an anti-tumor phenotype are potential therapeutic options for treatment of HPV-associated cancers.

Mechanisms of TAM-induced immune suppression in HPV^+^-associated cancers are not clearly understood (Figure 2C). It is assumed that the M2 polarization of macrophages leads to insufficient production of IL-12, a T cell-stimulating factor that is predominantly produced by M1 macrophages (144). IL-12 is required for the survival, differentiation and function of CTLs, Th1 cells and NK cells. Moreover, TAMs predominantly produce Th2-related cytokines such as IL-4, IL-10, and IL-13 (144), which promote Th2 cell polarization. The generation of Th2 cells forms a positive feedback loop to further stimulate TAM polarization and inhibit the response of other effector immune cells. In addition, TAMs play important roles in the recruitment of Tregs via M2-derived chemokines such as CCL22 (144). Treg activity can be further stimulated and maintained by high levels of IL-10 produced by TAMs. IL-10 may also promote the differentiation of naive CD4^+^ T cells toward Tregs and downregulate the function of DCs (144). Whether these mechanisms mediate immune suppression in HPV-associated cancers warrants further investigation.

Another example of regulatory innate leukocytes is myeloid-derived suppressor cells (MDSCs), which are a heterogeneous population of immature myeloid cells that consist of immature granulocytes, macrophages, and DCs with immunosuppressive functions. An increased frequency of MDSCs is associated with progression of different types of tumors (145). In studies of HPV-associated cancers, MDSCs were found in both blood and tumors in patients and were associated with a poor prognosis (146). Depletion of MDSCs in an HPV16-associated mouse tumor model enhanced the anti-tumor protection of a therapeutic vaccine targeting HPV16 E7 (147). The mechanisms underlying the expansion and activation of MDSCs in HPV-associated cancers remain unclear. Similar to TAMs, it is likely that MDSCs are stimulated by a variety of soluble factors, such as GM-CSF, VEGF, TNF-α, PGE2, IL-1β, IL-6, and IL-10, which are secreted by tumor and infiltrating immune cells, as well as tumor-associated fibroblasts (148) (Figure 2C). MDSCs can produce a broad range of suppressive molecules to inhibit the effector immune response (Figure 2C). They produce arginase 1 (Arg-1), inducible nitric oxide synthase (iNOS), IDO, reactive oxygen species (ROS) and immunosuppressive cytokines such as TGF-β and IL-10 (148). These molecules can act in either direct or indirect ways to inhibit survival and proliferation of effector T cells, inhibit NK cell function, and promote the recruitment and induction of Tregs. It has also been recently found that MDSCs upregulate PD-L1 expression (148), which can bind to PD-1 on the surface of effector T cells to suppress their activation. Due to the relevance of MDSCs in HPV-related carcinogenesis, several therapeutic approaches aiming to inhibit MDSC activity and recruitment are currently being tested in preclinical and clinical studies, and have shown beneficial effects in tumor growth inhibition and prolongation of survival (145, 149–152).

Overall, it is suggested that local microenvironment shaped by HPV infection plays a major role in modulating the phenotype and function of innate immune cells. These innate immune cells further produce mediators acting as a vicious cycle to maintain local immunosuppression. The connection among different innate immune cells plays an essential role in further regulating the adaptive immune response, which will be discussed below.

### Deregulation of Adaptive Immune Responses in HPV-Associated Cancers

#### CD8^+^ and CD4^+^ Effector T Cells

By interaction with mature APCs, naive CD8^+^ T cells can differentiate into CTLs which migrate to sites of infection to kill virus-infected cells. Infiltration of CD8^+^ T cells has been observed in HPV-associated tumors, yet they do not appear to prevent tumor growth (153). A mechanism by which the CTL response is inhibited might be associated with impairment of APC trafficking and maturation, as discussed above (Figure 2A). Enhanced CTL responses following DC maturation has been demonstrated in HPV16 E6 and E7-related mouse tumor models (154). Other factors might also be involved in the inhibition of CTL responses. For example, HPV-infected keratinocytes downregulate their surface MHC I expression (Figure 1D), thereby preventing CTL recognition (96, 155–157). Further studies on these possible mechanisms may unravel their contribution to HPV-induced immunosuppression.

In addition to a CTL response, a robust CD4^+^ T cell response is needed for the effective clearance of HPV. Patients with regressed HPV^+^ cervical lesions exhibited significantly higher HPV16 E6 and E7-specific CD4^+^ T cell responses compared to those with persistent CIN 1, 2, or 3 lesions (158, 159). Upon activation, naive CD4^+^ T cells have the potential to differentiate into various Th effector lineages, depending on which signals they receive from the local inflammatory environment. An anti-viral immune response requires a Th1 response, which is characterized by the secretion of the pro-inflammatory cytokines IFN-γ and IL-2 (160, 161). A lack of a Th1 response has been associated with persistent HPV infection and the development of high-grade disease (162–165). Interestingly, recent studies found that patients with high grade HPV^+^ cervical lesions had an increased Th2 response, characterized by the secretion of pro-tumorigenic cytokines such as IL-6, IL-8, and IL-10 (163). The shift from a Th1 toward a Th2 response might be, in part, responsible for HPV-immune evasion in the tumor environment (Figure 2D). Additionally, an IL-17-associated Th17 response has been recently described in patients with high grade HPV^+^ cervical lesions (166). A Th17 response is considered pro-inflammatory in the context of immune responses against extracellular pathogens but was found to be pro-tumorigenic in HPV-associated cancers. Furthermore, an increase of Th17 cell infiltration in HPV^+^ cervical lesions was associated with progression to invasive cervical cancer (167). The recruitment of Th17 cells may be mediated by tumor-derived chemokines. In the HPV16 E7 transgenic mouse model, the upregulation of a spectrum of chemokines was observed in E7 transgenic skin as a consequence of hyperproliferation, and chemokine receptor CXCR3 was found to promote T cell infiltration of skin. However, despite CXCR3 expression, T cells are ineffective in rejecting E7 transgenic skin when grafted onto non-transgenic mice (168). It is likely that Th17 cells in HPV^+^ cervical lesions play a similar role to these CXCR3^+^ T cells. Furthermore, a recent study suggests that the recruitment of Th17 cells is mediated by stromal tumor-associated fibroblasts (166). This observation provides new insights into the immune-modulating function of stromal cells in HPV-related carcinogenesis (Figure 2D). Finally, the recruitment of immunosuppressive cells such as Tregs was also observed in HPV^+^ cervical lesions and may further inhibit CTL function (169); Tregs will be discussed further below.

#### Regulatory T Cells

In persistent viral infections, the recruitment and activation of Tregs can promote viral immune evasion and contribute to disease pathogenesis. A few studies have analyzed Tregs in blood and tumors from high-risk HPV^+^ subjects, and revealed that patients with persistent HPV16 infection have a significantly higher CD4^+^ CD25^+^ Foxp3^+^ Treg frequency compared to those who have cleared the infection (159, 170). In addition, E6 and E7-specific Tregs were detected in both HPV-associated cervical tumors and tumor draining lymph nodes (171, 172). These HPV-specific Tregs were capable of suppressing proliferation and cytokine production from activated T cells *in vitro* upon stimulation with cognate HPV antigens.

Just how HPVs trigger the activation and expansion of Tregs is not clearly understood. Multiple factors in the local immune environment may act together (Figure 2D). Generally, the Treg response may be a result of the expansion of existing natural Tregs that are cross-reactive between self and HPV antigens. For example, the HPV16 E7 protein shares similarity with several human proteins such as retinoblastoma binding protein 1 (RBP-1) and XP-G complementing protein (XPGC) (173). Natural Tregs with self-reactivity to these self-proteins might be activated during HPV16 infection. Alternatively, natural Tregs can be stimulated in a non-specific way via their recognition of virus-associated molecular patterns as well as certain host products derived from damaged cells, such as heat shock proteins and β-defensins (174–176). Moreover, the Treg response may be a result of the expansion of induced Tregs that originate from either naive or differentiated CD4^+^ T cells. This usually requires T cell recognition of antigen presented by DCs in the presence of additional host-derived cytokines, such as TGF-β, IL-10, and IL-2 (177, 178). These cytokines are known to be upregulated in the HPV-associated tumor environment, where they are derived from a variety of local suppressive immune cells including TAMs, MDSCs, and Th2 cells, as discussed above. Furthermore, the induction of Tregs may be mediated by regulatory subsets of DCs (179, 180). Recent findings have suggested the presence of a regulatory type of APC in the dermal environment of HPV16 E7 transgenic mouse skin (124). The role of this DC subset in the induction of Tregs however, remains to be defined.

Immune suppression by Tregs is dependent on a variety of direct and indirect mechanisms. For example, Tregs can directly kill effector T cells via Fas-Fas ligand interactions (181) or via delivery of granzyme B and perforin (182). LAG-3 and CTLA-4 expressed on the surface of Tregs bind to MHC II and co-stimulatory molecules CD80/86, respectively, inhibiting the maturation of DCs and conditioning DCs to express regulatory molecules such as IDO (183, 184). In addition to direct contact-mediated inhibition, Tregs also elicit non-specific immunosuppressive effects by releasing a range of soluble mediators including the cytokines IL-10, TGF-β, and IL-35 (185–187). These cytokines are involved in inhibiting the proliferation and function of effector T cells and the maturation of DCs, as well as in inhibiting the pro-inflammatory activity of other innate immune cells. Moreover, Tregs express high levels of CD25, an essential component of the high affinity IL-2 receptor. This allows Tregs to compete with other activated T cells for the bioavailability of IL-2, thereby suppressing T cell survival and proliferation (188). Altogether, the mechanisms involved in Treg-mediated suppression are complex, and may vary between different stages of viral infection and the sites in which the immune response occurs. Our current understanding of Treg functionality is mainly established by *in vitro* studies. It is still unclear whether Tregs use the same mechanisms *in vivo*, and whether these mechanisms are applied by Tregs in the context of HPV-associated cancers.

Overall, infiltration of both CD8^+^ and CD4^+^ T cells has been observed in HPV-associated tumors, yet they are considered to be inefficient to eliminate HPV infected cells. Underlying mechanisms of their failure to eliminate HPV are not fully understood. However, current studies have indicated a shift from a Th1 toward a Th2 response in persistent HPV^+^ cervical lesions. In addition, patients with high grade HPV^+^ cervical lesions exhibit higher Th17 and Treg responses compared to those with regressed lesions. Collectively, these modulated immune responses might in part contribute to downregulation of CTL responses, and consequently HPV-immune evasion in the tumor environment.

### The Development of Immunotherapy for HPV-Associated Cancers

In the past few decades, there have been great advances in our understanding of the nature of HPV infection and its interaction with the host immune system. Based on these discoveries, different strategies have been trialed to prevent or treat HPV-associated diseases. HPV prophylactic vaccines and cervical screening have been introduced into prevention programs in many countries, which have been proven to be nearly 100% effective at preventing HPV-infection and HPV-associated cervical disease (189). However, prophylactic vaccines have limited utility in the treatment of existing HPV infections. For patients with pre-existing HPV-associated cancers, surgical removal with concomitant chemo- and/or radiotherapy remain the first choice of therapeutic intervention (190). However, several novel treatment options are becoming available to treat patients with more advanced disease (191). Immunotherapies are the most attractive among these novel treatment options, since they have potential to target persistent HPV infection in patients, which is the primary cause of disease.

### Current Status of Immunotherapies Against HPV

Current HPV prophylactic vaccines activate host immunity by stimulating the production of antibodies that specifically target HPV capsid proteins (192). However, these vaccines are incapable of eliminating HPV-infected cells, as capsid proteins are only present either before viral entry, or in the terminally differentiated epithelium. A therapeutic vaccine is therefore needed to elicit cell-mediated immune responses against viral antigens that are constitutively expressed in infected cells. Candidate viral targets that have been investigated include HPV early proteins such as E1, E2, E6, and E7 (193). To achieve the desired therapeutic outcome, therapeutic vaccines that deliver the viral antigens in several forms have been proposed and tested. These include live vector vaccines that use live attenuated bacteria or virus to deliver a recombinant vector expressing HPV antigens (194, 195). DNA vaccines are another powerful and economical vaccine platform. They consist of a DNA plasmid containing HPV genes of interest, which can be introduced to patients either via intramuscular or intradermal injections (196). Vaccines can also be made up of a mixture of HPV peptides which are known to be highly immunogenic (197), or recombinant HPV proteins that contain all potential antigenic epitopes (198, 199). Another attractive form of vaccine is a cell-based vaccine where, for example, autologous DCs derived from patients are cultured *in vitro* and stimulated with HPV peptides or proteins. These DCs are then transferred back to patients in order to present HPV antigens more efficiently to induce CTL responses (200). Many of these therapeutic vaccines have been tested for the treatment of HPV-associated diseases, although they have not demonstrated clinical success (201, 202). One of the concerns is that the induction and generation of HPV-specific CTLs alone by therapeutic vaccines might not be sufficient to eliminate HPV infected cells in patients with advanced diseases. This is probably because HPV-associated diseases employ multiple mechanisms to suppressive the immune system, as discussed above. Thus, an effective immunotherapeutic regimen for HPV patients will require a multi-pronged approach and deeper understanding of the underlying immunology.

Therapeutic vaccines are highly antigen-specific and therefore limited by their coverage of viral antigens and strains in each individual patient. An alternative option to stimulate immunity is the use of non-specific immune modulators that can potentially mediate positive anti-HPV immune responses regardless of HPV type. In many persistent viral infections and cancers, immune checkpoint molecules are over-expressed, leading to the lack of sufficient T cell responses for viral elimination. In some HPV-associated lesions and cancers, PD-1 expression on T cells was found to be significantly increased, compared with healthy controls (203, 204). Thus, it is reasonable to assume that the use of immune checkpoint inhibitors may improve anti-HPV immunity. Immune checkpoint inhibitors for clinical use, such as anti-CTLA-4 antibody (Ipilimumab) and human anti-PD-1 antibody (Pembrolizumab and Nivolumab), have been approved by U.S. Food and Drug Administration (FDA) to treat advanced melanoma patients (205). Multiple clinical trials have been designed and are being conducted on HPV-associated cancers (91).

Immune checkpoint inhibitors are designed to reduce the inhibitory signals in the tumor environment, while non-specific immune stimulants are used to augment the activation signals of immune cells. One example is the use of TLR agonists. TLR agonists are agents that mimic viral molecular patterns and therefore have the potential to boost TLR activation. For instance, Poly I:C mimics the structure of viral double stranded RNA, thus is an alternative stimulant of TLR3. The use of Poly I:C as a TLR3 agonist has been shown to activate DCs and promote E7-specific CTL responses in mice immunized with a HPV vaccine (206). Similar observations have also been shown in studies of TLR7 or TLR8 agonists (207), and Imiquimod (Aldara), a TLR7 agonist, is currently marketed for the treatment of anogenital warts caused by HPV (208). These results suggest that TLR agonists have potential to augment the DC response to viral antigens, thus promoting the activation of anti-HPV immunity.

### Future Prospects for the Immunotherapy of HPV-Associated Cancers

It is anticipated that the combination of different immunotherapeutic approaches will have the potential to overcome multiple barriers in HPV-mediated immunosuppression and thus restore an effective immune response to eliminate HPV-associated tumors. Some chemotherapeutic agents are good candidates to synergize with therapeutic vaccines, since they have immune-modulating effects through the enhancement of DC antigen presenting capability (209), the reduction of Treg numbers (210), and the depletion of circulating myeloid cells (211). In addition, the combination of therapeutic vaccines with immuno-regulatory antibodies or TLR agonists is being considered as a next-step therapeutic strategy (206). Moreover, to target immunosuppression in a more specific way, a new investigational vaccine strategy using the T-win^®^ technology has been recently described (212). These vaccines comprise long peptide epitopes derived from selected immunosuppressive molecules, such as metabolic enzymes (IDO and Arg-1), PD-L1, and the chemokine CCL22. It is expected that these vaccines will be able to activate endogenous T cells to directly target regulatory immune cells that highly express these immunosuppressive molecules. The T-win^®^ technology could synergize with current therapeutic vaccines in development, the potential of which has been tested in various mouse melanoma models (212). Several of these vaccines have been tested in phase I clinical trials and proved safe (212). They may prove to be a good immunotherapeutic choices for HPV-associated cancers in the future.

To support the development of immunotherapy, access to better research tools are needed. Recently, the development of high-throughput single-cell RNA sequencing techniques have allowed researchers to characterize the heterogeneity and interconnection among tumor cells, stromal cells, and immune cells within the tumor environment (213, 214). The analysis of gene expression profiles at single-cell resolution may also help identify new immunotherapeutic targets. To what extent this technique can help us understand immune evasion by HPV in the tumor environment will be an exciting topic over the next few years. To complement these research efforts, a mouse HPV-associated tumor model that more closely mimics human carcinogenesis needs to be developed. Current preclinical studies of therapeutic HPV vaccines are usually performed in mouse models inoculated with syngeneic tumor cells engineered to express HPV proteins, such as TC-1 cells (215). It is noted that TC-1 tumor progression and the associated immune environment might be different from those observed in human patients. Alternatively, mouse models that consistently express HPV16 oncoproteins on basal keratinocytes can better mimic the persistent high-risk HPV-related HSILs. For example, mice expressing the HPV16 E7 driven by keratin 14 promoter (K14E7) recapitulate the cellular and molecular profiles of HSILs in human subjects (216, 217), and therefore may be an appropriate model for studying the immune modulation by HPV oncoprotein and for developing and testing therapeutic vaccines. However, it has to be acknowledged that K14E7 transgenic models are not identical to HPV infection in humans, due to the lack of natural infection events. Moreover, these mouse models limit the choice of immune epitopes, and therefore might not be used to test those epitopes found exclusively in human patients. Recently, MHC-humanized mouse HPV tumor models have been developed (218), which could potentially be used as new vaccine testing platforms in the future. Nevertheless, the limitation of these humanized mouse models still exists as they are not able to mimic the entire human immune system. It is important to understand these limitations when interpreting data from these mouse models. This will avoid overestimating the immunogenicity and protective efficacy of therapeutic vaccines and therefore increase their translational success.

## Conclusion

HPV infection accounts for 30% of infection-related cancers worldwide and nearly 100% of cervical cancers in women. The causative role of HPVs has also been demonstrated in most anogenital cancers, whereas the potential etiological role of HPVs in non-anogenital cancers and skin cancers remains to be defined. The major risk factor for HPV-associated carcinogenesis is persistent infection of high-risk HPVs and their expression of E6 and E7 oncoproteins. These oncoproteins play essential roles in inducing cell immortalization. Moreover, they are able to modulate the host immune response by blocking immune-related gene expression and immune signaling pathways, as well as antigen presentation machinery in infected keratinocytes. The impairment of immune functions in keratinocytes further affects their communication with immune cells. This results in suppression of the overall anti-HPV immune response, characterized by inhibition of effector immune responses and enhancement of regulatory immune responses. Further studies are needed to understand the detailed mechanisms surrounding the crosstalk between cancer cells and different immune cells in the HPV-associated tumor environment. A better understanding of these interactions will help us develop new therapeutic strategies to overcome multiple barriers in HPV-mediated immunosuppression and synergize with current investigational therapeutic vaccines for treatment of HPV-associated cancers.

## Author Contributions

CZ contributed to the literature review and drafting of the manuscript. ZT and IF contributed to the drafting and critical revision of the manuscript.

### Conflict of Interest Statement

The authors declare that the research was conducted in the absence of any commercial or financial relationships that could be construed as a potential conflict of interest.
